# Somatic Cancer Driver Mutation Analysis in Endometriosis with Tumor-like Presentations: A Case Series Study

**DOI:** 10.3390/biomedicines14071636

**Published:** 2026-07-20

**Authors:** Lucy Chen, Elizabeth Severino, Dao-Sian Wu, Bhuchitra Singh, James Segars, Ie-Ming Shih

**Affiliations:** 1 Department of Gynecology and Obstetrics, Johns Hopkins School of Medicine, Baltimore, MD 21205, USA; lchen@ccrmivf.com (L.C.); elizabeth_severino@urmc.rochester.edu (E.S.); bsingh10@jhmi.edu (B.S.); jsegars2@jhmi.edu (J.S.); 2 College of Medicine, Taipei Medical University, Taipei 11031, Taiwan; albert93117@gmail.com; 3 Department of Pathology, Johns Hopkins School of Medicine, Baltimore, MD 21205, USA

**Keywords:** Endometriosis, cancer-driver mutations, pelvic pain

## Abstract

**Background/Objectives:** Endometriosis manifests as ectopic endometrial tissue outside the uterine cavity. This lesion can sometimes appear at unusual anatomical sites or within the intestinal tract, growing and mimicking cancer. Such tumor-like endometriosis lesions are relatively uncommon and biologically intriguing. **Methods:** This study is a retrospective case series of 14 patients presenting with tumor-like endometriosis at a single institution between 2007 and 2023. Laser capture microdissection was used to isolate epithelial cells from endometriotic glands in tissue sections from formalin-fixed, paraffin-embedded blocks, and the microdissected epithelium was analyzed for cancer driver mutations. **Results:** Unlike conventional endometriosis, these tumor-like lesions were generally sizable and clinically presented in the groin area, aortic wall, omentum, bowel wall, or lymph nodes, all of which raised suspicion for malignancy, despite 11 (78.6%) of the 14 cases having a history of or clinical signs of endometriosis. A total of 11 cancer-driver mutations were identified in 6 of the 14 patients, with four patients harboring multiple mutations. Recurrent mutations included *KRAS*-activating mutations in four cases and *ARID1A*-inactivating mutations in two cases. Additional mutations involved *PIK3CA*, *CTNNB1*, *CHD4*, *MYD88*, and *STAG2*. **Conclusions:** This study detected mutations in *KRAS*, *ARID1A*, *PIK3CA*, *CTNNB1*, and *MYD88* in endometriotic lesions, consistent with previous literature, and identified newly reported mutations in *CHD4* and *STAG2*. Not every tumor-like lesion harbored cancer driver mutations.

## 1. Introduction

Endometriosis is a reproductive disorder characterized by the growth of endometrial tissue outside the uterus, affecting about 10% of women of reproductive age worldwide [1]. Chronic pelvic pain and infertility are two primary clinical symptoms [1,2,3]. Endometriosis is traditionally classified into three subtypes based on location: superficial endometriosis, deep infiltrating endometriosis (DIE), and ovarian endometriotic cysts or endometriomas [2]. Ovarian endometriosis has also been identified as a precursor to ovarian clear cell and endometrioid carcinomas [4,5].

The mechanism of development of the three subtypes of endometriosis is an ongoing area of investigation. Sampson’s implantation hypothesis explains the development of endometriosis as a consequence of retrograde menstruation of endometrial tissue through the fallopian tubes [1]; however, this theory does not explain why some lesions remain superficial, some become endometriomas, and some become deeply invasive. Recent molecular genetic studies suggest a modified paradigm for how DIE forms, where circulating epithelial progenitor or stem cells that are intended to rebuild the uterine endometrium after menstruation become excessively reactive and then malpositioned outside the uterus [2]. These entrapped epithelium-committed progenitor cells produce new glands through clonal proliferation and recruit oligoclonal stromal cells, resulting in the formation of DIE. Once formed, the ectopic tissue is exposed to immune surveillance and reaction, leading to inflammation and fibrosis, causing chronic pain, infertility, and other gastrointestinal symptoms [6,7,8].

While most cases of endometriosis involve the ovaries, fallopian tubes, peritoneal surface, bladder, and bowel serosa, some cases are more widely spread throughout the body. These rare instances can appear in unusual locations and grow as masses that may be mistaken for a neoplastic process on imaging studies. Such cases have been reported in various sites, including the groin, diaphragm, umbilicus, brain, nasal cavity, skeletal muscle, liver, pancreas, lung, kidney, and biliary tract [9,10,11,12]. Here, the term “tumor-like” endometriosis is used to describe those.

Cancer driver mutations such as *KRAS*, *ARID1A*, *PIK3CA*, *CTNNB1*, *MYD88*, *PPP2R1A*, *FGFR2*, and *PTEN* have been detected and are implicated in the pathology of endometriosis [13,14,15], endometriosis-associated ovarian cancer [16], or endometrioid carcinoma [17,18,19,20,21,22]. These shared mutations between uterine and ovarian cancer and endometriosis are an active area of investigation. To begin characterizing the biological and pathological features of tumor-like endometriosis cases, this study aimed to identify somatic sequence mutations in cancer-driver genes within the laser-captured micro-dissected glandular epithelium from 14 lesions. Characterization of cancer-driver mutations in samples from tumor-like endometriosis may help elucidate the biologic mechanisms underlying their growth and ability to arise outside conventional anatomic locations.

## 2. Materials and Methods

### 2.1. Sample Selection

This retrospective case series of tumor-like endometriosis included women treated at the Johns Hopkins Medical Institution from 2007 to 2023. The institutional review board approved this study under IRB00188126. Cases included in this report were endometriotic lesions outside conventional pelvic locations (e.g., peritoneal surface, endometriomas, or deeply invasive endometriosis of the pelvic sidewall), such as cases involving lymph nodes, infiltration into the muscularis or submucosa of the gastrointestinal tract, and other unusual sites. These cases were originally suspicious for neoplasms based either on clinical impression or imaging studies. Hematoxylin and eosin slides were reviewed, and the diagnosis of endometriosis was confirmed by two authors (LC and IS) following the criteria in diagnostic pathology [23]. Cases were excluded if they had inadequate tissue samples for laser capture microdissection or any evidence of neoplastic or precancerous disease of the female reproductive tract. Clinical and demographic information was obtained through the electronic medical record, and formalin-fixed, paraffin-embedded (FFPE) tissues from qualified cases were retrieved from archival files. All cases were subsequently anonymized, and no patient health information could be retrieved or identified.

### 2.2. Preparation of Tissue for Whole-Exome Sequencing

Protocols for preparation of DNA from laser-capture microdissected epithelial cells were used as previously reported [24], and whole-exome sequencing, including somatic mutation calling, followed protocols previously used in the literature [25,26]. Briefly, FFPE tissue blocks were sectioned at 10 μm and mounted on PEN membrane slides (Zeiss, Oberkochen, Germany). Slides were deparaffinized with xylene and ethanol baths and then stained with hematoxylin. Targeted tissues were dissected using a Leica LMD7 laser-capture microdissection microscope to enrich for the epithelial component of ectopic endometrial glands. Adjacent non-endometriotic tissue, such as smooth muscle, was collected as a germline control. Genomic DNA was extracted with Qiagen’s QIAamp DNA FFPE Tissue Kit (Qiagen, Germantown, MD, USA). DNA concentration was measured with a Qubit dsDNA HS Assay on a Qubit 2.0 Fluorometer (Life Technologies, Carlsbad, CA, USA). gDNA was fragmented into 150–200 base pair pieces.

Whole-exome sequencing was performed using the Illumina NovaSeq 6000 platform (Illumina, San Diego, CA, USA), and data of matched lesion/tumor and normal samples were aligned to the human reference genome (hg38) using BWA software and analyzed to identify somatic point mutations and small insertions and deletions present in the lesion/tumor but not in matched normal samples. This study defined cancer-driver genes following guidelines and prediction algorithms as previously reported [27]. The functional consequence of each mutation was predicted using gene annotations with ANNOVAR 2018Apr16, using databases from SIFT, Polyphen 2HDIV prediction, and MutationTaster prediction. This study focused solely on detecting DNA sequence variations in the cancer driver genes from the glandular epithelium. After identifying somatic mutations in cancer driver genes, all sequencing data were deleted in conformity with the IRB protocol.

### 2.3. Whole Exome Sequencing Data Analysis

Somatic variants were identified using a customized pipeline following GATK Best Practices. Sequencing reads from Novaseq underwent quality control, adapter and low-quality base trimming (FastQC, Trimmomatic), and alignment to hg38 (BWA). Duplicates were marked (Picard), indel regions realigned, and base scores recalibrated (GATK BQSR). We filtered nonsynonymous single nucleotide variants, splice site variants, and indels in coding regions of cancer driver genes according to the following criteria: tumor sample coverage ≥ 10X; normal sample coverage ≥ 8X; variant allele frequency ≥ 0.1; normal frequency ≤ 0.02; normal frequency/tumor frequency < 0.2. Variants were called with MuTect and Strelka and annotated using VEP and Oncotator. Filtering retained only high-confidence nonsynonymous, splice site, and coding indels based on coverage and allele frequency thresholds. Manual review (IGV) and public databases (dbSNP, 1000 Genomes, ESP, ExAC) excluded common germline variants. Non-synonymous mutations were classified as drivers if supported by the Cancer Gene Census or literature and as passengers if lacking evidence for involvement in cancer. In this study, we only focus on reporting mutations in known cancer driver genes.

### 2.4. Statistical Analysis

This study is primarily descriptive due to the rarity of cases and limited sample size.

## 3. Results

Table 1 summarizes the overall clinical and demographic data for all 14 cases in this cohort. The mean age at the time of surgery to remove tumor-like endometriosis was 39.6 years, and the mean BMI was 29.1. As expected, most cases (85.7%) presented as stage IV disease, and 57.1% of patients had a history of previous surgery for endometriosis. Uterine leiomyoma or adenomyosis was found in every patient. Two-thirds of the patients were nulliparous.

The table below (Table 2) lists the clinical features, treatment histories, and cancer driver mutations for individual cases. Variant allele frequency in those cancer driver genes listed in Table 2 were all above 0.1, ranging from 0.12 to 0.30 (average 0.16), depending on the levels of epithelial cell enrichment after microdissection and the presence of intra-epithelial lymphocytes. Cases 1 to 5 showed distant involvement, manifesting as an umbilical nodule, a groin mass, a large lesion encroaching the aorta, lymph node enlargement, and an omental mass, respectively. Four cases (cases 11–14) presented with appendiceal endometriosis together with other concurrent endometriotic lesions: three of the four cases were found to have concomitant ovarian endometriosis. Gastrointestinal endometriosis was found in six cases, showing deep infiltration of endometriosis into the muscularis and submucosa of the bowel wall.

Case 1 involves a 45-year-old woman who was evaluated for surgical removal of a solitary subcutaneous nodule at the umbilicus. Her symptoms started 15 years ago, with the lesion gradually becoming increasingly swollen, painful, and bloody during menstruation. The clinical impressions included skin appendage neoplasia and endometriosis. She underwent excision of her umbilical nodule to diagnose endometriosis, which was also found to affect the anterior cul-de-sac, uterosacral ligaments, and ovarian tissue. A primary umbilical hernia complicated her postoperative recovery. She was not found to have any cancer driver mutations in this umbilical endometriosis.

**Figure 1:**
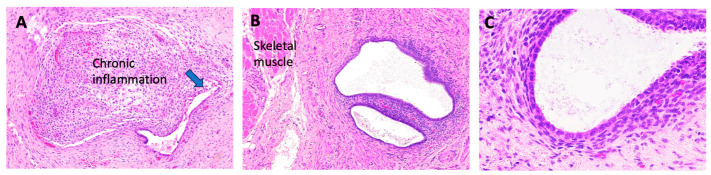
An inguinal mass containing endometriosis shows hematoxylin and eosin-stained morphological features from case 2. (**A**) At 10× magnification, the lesion shows focal chronic inflammation, possibly due to injury to the endometrial gland (arrow). (**B**): At 20× magnification, both glands and stroma are evident and are surrounded by fibrosis. Skeletal muscle is also shown. (**C**): At 40× magnification, the endometriosis is composed of both glandular and stromal components.

Case 2 involves a 35-year-old woman with a past medical history of infertility and prolactinoma who was initially evaluated for a right inguinal mass suspected to be a desmoid tumor. The mass had been present for 10 years, and she reported that it became prominent and tender during menstruation. MRI detected a right inguinal mass in the subcutaneous fat alongside a left para-ovarian cyst. The pathology report after excision revealed florid endometriosis with focal chronic inflammation (Figure 1). She had no history of cesarean section or other gynecologic surgeries. Her inguinal endometriosis harbors a *KRAS* mutation.

**Figure 2:**
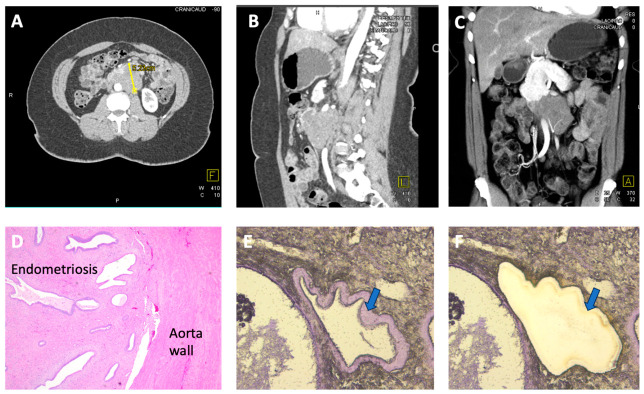
A peri-aortic tumor-like endometriosis. (**A**–**C**): Computed tomography scan reveals a 6.3 cm retroperitoneal mass involving the abdominal aorta from case 3. (**D**): Hematoxylin and eosin-stained section shows haphazardly distributed endometrial glands embedded in dense fibrous tissue involving the aortic wall. (**E**): An endometrial gland (arrow) before laser capture microdissection at 40× magnification. (**F**): The same gland (arrow) after microdissection at 40× magnification. The epithelial layer has been collected for analysis.

Case 3, a 49-year-old woman, had a history of endometriosis for which she underwent hysterectomy and bilateral oophorectomy. Nine years later, she developed abdominal pain and bowel obstruction. Her abdominal CT scan showed a slowly enlarging retroperitoneal mass involving the abdominal aorta, initially suspected to be an abdominal aneurysm or a retroperitoneal soft tissue tumor (Figure 2). Subsequently, she underwent exploratory laparotomy with *en bloc* resection of the large retroperitoneal mass, which was surprisingly identified on pathologic analysis as an endometriotic mass. She was found to have mutations in *CTNNB1* and *ARID1A* in this retroperitoneal endometriosis involving the aorta.

**Figure 3:**
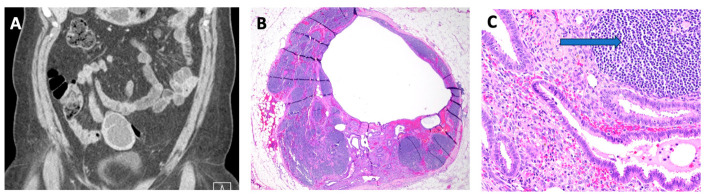
An endometriotic lesion found in a bowel lymph node, forming an enlarged pelvic mass in case 4. (**A**): Computed tomography scan shows an enlarged lymph node near the colon. (**B**): Hematoxylin and eosin-stained section at 4× magnification reveals dilated endometrial glands enlarging this lymph node. (**C**): Hematoxylin and eosin-stained section at 20× magnification displays endometrial glands surrounded by stromal cells. A lymphoid follicle (arrow) is adjacent to the endometriotic tissue.

Case 4, a 49-year-old woman, developed a right-sided ovarian mass and left ureteral obstruction requiring percutaneous nephrostomy. Her abdominal and pelvic CT scans showed a right ovarian lesion with suspicious features concerning for neoplastic disease. Pathologic analysis revealed a right endometriotic ovarian cyst and evidence of endometriosis within an enlarged and engorged left pelvic sidewall lymph node and a portion of the sigmoid colon. These samples also demonstrated evidence of adenomyosis. The imaging and pathology of the involved lymph node are shown in Figure 3. Because lymph node endometriosis is less common, the epithelium was examined, and no cancer driver mutations were identified.

Case 5 involves a 28-year-old woman with a history of cervical atresia, infertility, severe endometriosis with dysmenorrhea, and known homozygous *MTHFR* germline mutations. She developed extensive adhesions among the pelvic organs, including the uterus, rectum, sigmoid colon, ovaries, fallopian tubes, and pelvic sidewalls. During hysterectomy and bilateral salpingo-oophorectomy, an omental lesion was discovered. Her pathology report indicated omental endometriosis with no cancer driver mutations. 

Case 6, a 39-year-old woman, presented with one week of abdominal pain and vomiting. The clinical impression included possible small bowel obstruction, cholecystitis, gastritis, abdominal aortic aneurysm, or gastroenteritis. She underwent an exploratory laparotomy and the pathology report revealed extensive endometriosis involving all layers of the wall of the sigmoid colon. Evidence of endometriosis was found in a pelvic lymph node, on the mesentery, and within the abdominal wall, the colonic serosa, muscularis, and submucosa. The sigmoid colon endometriosis lesion was found to have somatic mutations in *CHD4*, *MYD88*, and *STAG2*.

Case 7 was a 17-year-old female with a history of constipation. On exam, she had a well-defined, non-tender, firm right lower quadrant mass that was rapidly enlarging. Pathology showed bilateral endometriotic cysts and endometriosis involving the rectum (serosa, muscularis, and submucosa) and the omentum. Her omental endometriosis did not show any cancer driver mutations.

Case 8 involved a 39-year-old presenting with a bowel obstruction. Ultrasound and MRI revealed a large, complex cystic mass, with differential diagnoses including a large endometrioma and ovarian carcinoma. She underwent an exploratory laparotomy with bilateral oophorectomy, ovarian cystectomy, extensive ureterolysis, ileocecal resection, and reanastomosis. The pathology report confirmed endometriotic cysts on both sides and extensive endometriosis with transmural involvement of the rectosigmoid colon and ileocecum. The rectosigmoid endometriosis harbored mutations in *KRAS* and *ARID1A*.

Case 9 involved a 51-year-old presenting with abnormal findings on a barium enema and an inability to pass sigmoidoscopy. The pathology report showed transmural endometriosis of the sigmoid colon, leading to bowel obstruction. No cancer driver mutations were found in her sigmoid endometriosis.

Case 10 involved a 46-year-old who presented with complications following a robotic hysterectomy performed at an outside hospital due to endometriosis. Segment resection of her colon revealed focal endometriosis involving the muscularis propria and pericolonic fibroadipose tissue. Her colonic endometriosis was found to have a *KRAS* mutation.

Case 11 presented at age 34 with menses-associated nausea, vomiting, and diarrhea. Abdominal and pelvic CT scans showed a dilated appendix and a right adnexal cyst. Pathology of the appendectomy specimen revealed extensive endometriosis involving the muscularis propria and adipose tissue of the appendix. Nucleotide sequencing of the endometriosis lesion did not detect any cancer driver mutations.

**Figure 4:**
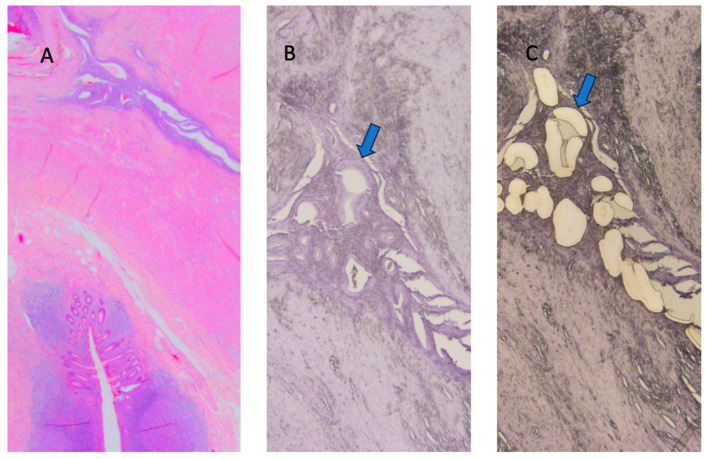
Appendiceal endometriosis from case 12. (**A**): Hematoxylin and eosin-stained section at 4× magnification shows endometriosis involving the peri-appendiceal soft tissue with extensive fibrosis and smooth muscle proliferation. (**B**): Endometrial glands (arrow) at 10× magnification before laser capture microdissection. (**C**): Same glands after microdissection at 10× magnification. The epithelial layer has been collected for analysis.

Case 12 presented at age 41 with dyschezia during menses. She had undergone diagnostic laparoscopy, which revealed an appendiceal mass suspicious for appendiceal neoplasm. Pathology findings showed endometriosis of the appendix with extensive involvement of the peri-appendiceal soft tissue, resulting in fibrosis and accounting for the tumor-like lesion (Figure 4). Endometriosis also involved the serosal surface of the uterus and the right fallopian tube. Her pain improved after surgical removal of the appendix, uterus, and fallopian tubes. Her appendiceal endometriosis showed *KRAS* and *PIK3CA* mutations.

Case 13 involved a 31-year-old woman who presented with chronic pelvic pain and cyclic small bowel obstructions. She underwent an ileocolic resection that revealed endometriosis affecting the ileal serosa, causing stricture formation, serosal adhesions, and adhesion of the appendix. Her endometriosis did not contain any cancer driver mutations.

Case 14 involved a 55-year-old woman who was found to have endometriosis incidentally during surgery for metastatic neuroendocrine cancer. She had multiple foci of endometriosis involving the serosa of the ileum and both the wall and serosa of the appendix. Her appendiceal endometriosis did not demonstrate evidence of any cancer driver mutations.

## 4. Discussion

This study reported clinical and molecular genetic findings of “tumor-like endometriosis” to explore the biological nature of these unusual lesions, which appear in uncommon anatomical locations, including lymph nodes and intestinal endometriosis, mimicking neoplastic diseases. The findings from this study are expected to have several clinical and biological implications.

First, there did not appear to be a specific age group associated with increasing tumor-like endometriosis, as ages at clinical presentation ranged from 17 to 51 years, indicating that tumor-like endometriosis can occur at any age. Second, patients in this cohort experienced varying levels of treatment success with progesterone and NSAIDs for the management of pain and gastrointestinal symptoms. Pain continued despite surgical intervention in three patients. As with more typical presentations of endometriosis, response to intervention varies widely even amongst relatively clinically similar endometriotic lesions, the reason for which has yet to be elucidated.

Third, endometriosis in unusual anatomical locations is of particular interest. Umbilical and groin endometriosis lesions (case 1 and case 2) represent classic examples of extrapelvic endometriosis. These lesions are considered uncommon, yet they have been documented in a significant number of cases [10]. Involvement of the aorta, reported as case 3 here, is probably the second-best-known case in the literature [28]. For individuals with endometriosis in rare anatomic locations, increased knowledge and clinical vigilance about the disease, along with a multidisciplinary approach, are recommended to ensure timely and accurate diagnosis. Additionally, among the 14 cases, two showed lymph node endometriosis, resulting in nodal enlargement. Although uncommon, lymph node endometriosis has been documented in the literature. Most of these lesions involve the mesentery and pelvic lymph nodes, but the para-aortic obturator node has also been reported [29]. The presence of endometriosis in lymph nodes suggests that endometriosis may spread via lymphatic pathways rather than only through direct local dissemination. The occurrence of appendiceal endometriosis in four cases supports the idea that these lesions are not uncommon. One study involving patients undergoing laparoscopic endometriosis surgery at a tertiary referral center estimated the prevalence of appendiceal endometriosis at 2.8%, with a higher risk observed in women with ovarian and bladder endometriosis [30].

Fourth, our results emphasize that several somatic cancer-driver mutations are of great interest in the study of endometriosis because these mutations and the pathways they affect may play a role in the disease’s development. The original study that identified cancer driver mutations in endometriosis found that 26% of deep-infiltrating endometriotic lesions contained cancer-causing mutations in the endometriotic epithelium, including *KRAS*, *PIK3CA*, *ARID1A*, and *PPP2R1A*, and proposed a clonal origin for endometriosis [18]. Uterine endometrioid carcinomas and endometriosis-related ovarian malignancies typically exhibit these gene mutations [31]. Moreover, these mutations are also detected in the precursor lesions of uterine endometrioid carcinomas [25], supporting the idea that (ovarian) endometriosis may predispose to ovarian endometrioid or clear cell carcinomas.

Most importantly, our data showed that the overall mutation frequency of cancer-driver genes in our cohort was comparable to that in other endometriosis cohorts in the literature, including superficial, deeply infiltrating, and endometriomas [18,19,20,21,22]. In light of the tumor-promoting functions of somatic mutations in cancer-associated genes [32], this finding may be surprising. This is because endometriosis consists of non-neoplastic tissues, exhibits minimal proliferation, and is histologically indistinguishable from eutopic endometrium. It remains unclear whether these or other cancer-driver mutations are involved in the development of tumor-like endometriosis in some cases within this cohort. Several explanations for why tumor-like endometriosis lesions do not show a higher frequency of cancer-driver mutations include the following. The specific co-occurrence of mutations in cancer-driver genes is important for tumor formation. For instance, concurrent inactivation of the tumor suppressors *ARID1A* and *PTEN* is necessary to increase proliferation in endometrioid intraepithelial neoplasia, the immediate precursor lesion of the endometrium [33], and to induce endometrioid carcinoma in a mouse model [34], likely through activation of the MAPK signaling via DUSP4 downregulation [35]. However, co-mutation of *ARID1A* and *PTEN* was not observed in this cohort.

The next interpretation is that mutations in these genes may have functions beyond carcinogenesis. For example, studies show that increased activity of the KRAS signaling pathway, whether through genetic or epigenetic mechanisms, may promote the survival of ectopic endometrium and contribute to progesterone resistance. In mouse models, activation of the KRAS pathway was associated with endometriosis-like lesions on the peritoneum and ovaries [36], and endometriosis lesions from mice with activating Kras mutations exhibited prolonged survival compared with those in wild-type mice [37]. A separate study found that KRAS pathway activation caused abnormal overexpression of *SIRT1*, which co-localizes with BCL6, thereby promoting progesterone resistance by inactivating the *GLI1* promoter [38]. In a retrospective longitudinal study, mutations in *KRAS* were associated with higher disease severity and surgical difficulty [22].

Another possible explanation is that these cancer-driver mutations may serve as clonal markers associated with their development, with less biological significance. Due to the absence of a parallel assessment of eutopic endometrium in our study, it remains undetermined whether the mutation rate identified among tumor-like endometriosis is greater than that of eutopic endometrium in our cohort. It has been reported that individual endometrial glands and micro-dissected tissues from normal uterine endometrium share a similar set of somatic cancer-associated mutations as those found in endometriosis [19,39]. From this perspective, normal endometrial glands may undergo clonal expansion, carrying specific mutations in epithelial cells that remain in situ. These glands then exit the uterine cavity through retrograde menstruation or spread via circulation, leading to endometriosis [2,40]. From this perspective, cancer-associated mutations are considered indolent and occur alongside the growth of endometriotic lesions that harbor mutations.

Lastly, this study detected several mutations that have previously been identified within endometriotic tissue, including *KRAS*, *ARID1A*, *PIK3CA*, *CTNNB1*, and *MYD88.* Two novel somatic mutations were identified in genes not previously reported in endometriosis, namely *CHD4* and *STAG2*. Further studies are necessary to characterize their roles in the disease process. The SNF2/RAD54 helicase family includes the transcriptional repressor *CHD4*. Mutations in these genes have been observed in endometrial carcinomas and their precancerous lesions [25,41]. The *CHD4*, *R975H* mutation is linked to endometrial cancer cell stemness and M2-like polarization in tumor-associated macrophages [41]. On the other hand, *STAG2*, a tumor suppressor, is a core protein in the cohesin complex, responsible for chromosome segregation during cell division, DNA repair, gene expression, and prevention of telomere recombination [42]. *ARID1A* or *STAG2* mutations may cause telomere cohesion problems, the effects of which on the pathogenesis of endometriosis warrant further study. Functional analysis of the impact of these mutations on transcriptomics and protein function was outside the scope of this study but is an important topic for future work.

Despite the new insights gained from this study, several limitations are also recognized. The small sample size of this case series limits the statistical power to conduct a correlational study between somatic mutation status and clinical parameters. Because tumor-like endometriosis is relatively rare, a future collaborative effort is necessary to clarify its associations. Additionally, all included patients were undergoing surgical intervention at a single center, which could introduce selection bias. Moreover, DNA quality is known to be affected by the age of tissue blocks, as samples become more fragmented, and the quantity is also limited by laser capture microdissection. Although this did not impact the identification of cancer driver mutations, it may lead to under-detection of mutations that were not enriched in the epithelium of endometriosis. 

## 5. Conclusions

*CHD4* and *STAG2* are cancer driver mutations detected in endometriotic lesions in this study that have not previously been reported to be associated with endometriosis. Mutations in *KRAS*, *ARID1A*, *PIK3CA*, *CTNNB1*, and *MYD88* were also identified, which have previously been reported in the setting of endometriosis. Cancer driver mutations were not present in all samples of tumor-like endometriosis, implying that cancer driver mutations are not the sole promoters of extreme presentations of endometriosis and that they may have other physiological functions in endometriosis beyond their canonical tumor-promoting roles.

## Figures and Tables

**Table 1:** Demographic and clinical characteristics of a cohort of patients with tumor-like endometriosis.

Tumor-like Endometriosis Characteristics (*n* = 14)	
Race, *n* (%)	
White	4 (28.57)
Asian	3 (21.43)
African American	6 (42.86)
Other	1 (7.14)
ASRM stage, *n* (%)	
Stage I	0 (0)
Stage II	2 (14.29)
Stage III	0
Stage IV	12 (85.71)
Age at surgery (y), mean (SD)	39.64 (9.32)
BMI at surgery (kg/m^2^), mean (SD)	29.13 (7.69)
Other gynecological conditions, *n* (%)	
Fibroids, *n* (%)	8 (57.14)
Adenomyosis, *n* (%)	5 (35.71)
Pregnancy history, *n* (%)	
Nulliparous	9 (64.29)
Parous	5 (35.71)
Smoking history, *n* (%)	
Reports smoking	4 (28.57)
Does not report smoking	10 (71.43)
History of medical treatment for endometriosis, *n* (%)	4 (28.57)
History of prior surgical management of endometriosis, *n* (%)	8 (57.14)

**Table 2:** Case descriptions.

Case Number	Lesions	Presenting Symptom(s)	Stage	CDM(s)	Hormonal Treatment	Surgical Treatment	Other GYN Conditions	Outcome
1	Umbilical nodule	Dysmenorrhea	2	None	Yes	No	Fibroids	Repeat surgery
2	Groin	Chronic groin pain	4	*KRAS* (c.35G>A; p.Gly12Asp)	No	No	None	Pursuing infertility treatment
3	Aorta	Chronic pelvic pain	4	*CTNNB1* (c.599G>A; p.Arg200His), *ARID1A* (c.4336C>T; p.Arg1446Term)	No	Yes	Fibroids	Lost to follow-up
4	LN, colonic wall	Chronic pelvic pain, dysmenorrhea	4	None	No	No	Fibroids, Adenomyosis	Pain improved
5	Omentum	Chronic pelvic pain, dysmenorrhea	4	None	Yes	No	Adenomyosis	Lost to follow-up
6	Sigmoid colonic wall, LN	Dysmenorrhea, constipation, SBO	4	*CHD4* (c.2924G>A; p.Arg975His), *MYD88*, (C.71C>T; P.ALA24VAL) *STAG2* (c.3467+1G>A)	Yes	Yes	Adenomyosis	Pain improved
7	Omentum and rectal wall	Dysmenorrhea, constipation	4	None	No	No	None	Lost to follow-up
8	Rectosigmoid colonic wall	Chronic pelvic pain, SBO	4	*KRAS* (c.34G>T; p.Gly12Cys), *ARID1A* (c.197C>T; p.Pro66Leu)	No	Yes	Fibroids, Adenomyosis	Controlled by progestin
9	Sigmoid colonic wall	SBO, chronic pelvic pain	4	None	No	Yes	Fibroids, Adenomyosis	Post-menopausal after surgery
10	Colonic wall	Chronic pelvic pain	4	*KRAS* (c.35G>C; P.Gly12Ala)	No	Yes	Fibroids	Concern for recurrence
11	Appendix	Chronic pelvic pain	4	None	No	Yes	Fibroids	Pain improved
12	Appendix	Chronic pelvic pain, dyschezia	2	*KRAS* (c.35G>T; p.Gly12Val), *PIK3CA* (c.1035T>A; Asn345Lys)	No	Yes	Fibroids	Lost to follow-up
13	Appendix	Chronic pelvic pain, nausea, vomiting, SBO	4	None	Yes	Yes	None	Pain continued, controlled by progestin
14	Appendix	Chronic pelvic pain	4	None	No	No	None	Lost to follow-up

Abbreviations: CDM, Cancer Driver Mutation (specifically referring to somatic mutations within endometriotic foci that were not present in control/germline tissue, see Methods section for details); SBO, Small Bowel Obstruction; LN: lymph node. ‘Lost to follow-up’ means that no clinical information was present in the EMR after surgical intervention. The gray shading helps visualization.

## Data Availability

The data presented in this study are available on request from the corresponding author. The larger repository of exome data has been deleted; specific mutation-level data is available upon request. Remaining data, including demographic and clinical information without PHI, is available upon request.

## References

[1] Bulun S.E. (2009). Endometriosis. N. Engl. J. Med..

[2] Wang Y., Nicholes K., Shih I.M. (2020). The Origin and Pathogenesis of Endometriosis. Annu. Rev. Pathol..

[3] Simoens S., Dunselman G., Dirksen C., Hummelshoj L., Bokor A., Brandes I., Brodszky V., Canis M., Colombo G.L., DeLeire T. (2012). The burden of endometriosis: Costs and quality of life of women with endometriosis and treated in referral centres. Hum. Reprod..

[4] Wilbur M.A., Shih I.M., Segars J.H., Fader A.N. (2017). Cancer Implications for Patients with Endometriosis. Semin. Reprod. Med..

[5] Chui M.H., Wang T.L., Shih I.M. (2017). Endometriosis: Benign, malignant, or something in between?. Oncotarget.

[6] Herington J.L., Bruner-Tran K.L., Lucas J.A., Osteen K.G. (2011). Immune interactions in endometriosis. Expert Rev. Clin. Immunol..

[7] Ahn S.H., Khalaj K., Young S.L., Lessey B.A., Koti M., Tayade C. (2016). Immune-inflammation gene signatures in endometriosis patients. Fertil. Steril..

[8] Sisnett D.J., Zutautas K.B., Vo D.H.N., Ravishanker K., Holmes J.P., Wodz A., Tayade C. (2026). Immune dysregulation in endometriosis: The T cell perspective. Front. Immunol..

[9] Victory R., Diamond M.P., Johns D.A. (2007). Villar’s nodule: A case report and systematic literature review of endometriosis externa of the umbilicus. J. Minim. Invasive Gynecol..

[10] Andres M.P., Arcoverde F.V.L., Souza C.C.C., Fernandes L.F.C., Abrao M.S., Kho R.M. (2020). Extrapelvic Endometriosis: A Systematic Review. J. Minim. Invasive Gynecol..

[11] Dalkalitsis A., Salta S., Tsakiridis I., Dagklis T., Kalogiannidis I., Mamopoulos A., Daniilidis A., Athanasiadis A., Navrozoglou I., Paschopoulos M. (2022). Inguinal endometriosis: A systematic review. Taiwan. J. Obstet. Gynecol..

[12] Arakawa T., Ishihara S. (2021). Omental Endometriosis. N. Engl. J. Med..

[13] Grechukhina O., Petracco R., Popkhadze S., Massasa E., Paranjape T., Chan E., Flores I., Weidhaas J.B., Taylor H.S. (2012). A polymorphism in a let-7 microRNA binding site of KRAS in women with endometriosis. EMBO Mol. Med..

[14] Hossain M.M., Nakayama K., Shanta K., Razia S., Ishikawa M., Ishibashi T., Yamashita H., Sato S., Iida K., Kanno K. (2021). Establishment of a Novel In Vitro Model of Endometriosis with Oncogenic KRAS and PIK3CA Mutations for Understanding the Underlying Biology and Molecular Pathogenesis. Cancers.

[15] Su W., Cui H., Wu D., Yu J., Ma L., Zhang X., Huang Y., Ma C. (2021). Suppression of TLR4-MyD88 signaling pathway attenuated chronic mechanical pain in a rat model of endometriosis. J. Neuroinflamm..

[16] Wiegand K.C., Shah S.P., Al-Agha O.M., Zhao Y., Tse K., Zeng T., Senz J., McConechy M.K., Anglesio M.S., Kalloger S.E. (2010). ARID1A Mutations in Endometriosis-Associated Ovarian Carcinomas. N. Engl. J. Med..

[17] Zyla R.E., Olkhov-Mitsel E., Amemiya Y., Bassiouny D., Seth A., Djordjevic B., Nofech-Mozes S., Parra-Herran C. (2021). CTNNB1 Mutations and Aberrant β-Catenin Expression in Ovarian Endometrioid Carcinoma: Correlation with Patient Outcome. Am. J. Surg. Pathol..

[18] Anglesio M.S., Papadopoulos N., Ayhan A., Nazeran T.M., Noe M., Horlings H.M., Lum A., Jones S., Senz J., Seckin T. (2017). Cancer-Associated Mutations in Endometriosis without Cancer. N. Engl. J. Med..

[19] Suda K., Nakaoka H., Yoshihara K., Ishiguro T., Tamura R., Mori Y., Yamawaki K., Adachi S., Takahashi T., Kase H. (2018). Clonal Expansion and Diversification of Cancer-Associated Mutations in Endometriosis and Normal Endometrium. Cell Rep..

[20] Li L., Antero M.F., Zhang M., Chu T., Seckin T., Ayhan A., Pisanic T., Wang T.L., Cope L., Segars J. (2021). Mutation and methylation profiles of ectopic and eutopic endometrial tissues. J. Pathol..

[21] Lac V., Verhoef L., Aguirre-Hernandez R., Nazeran T.M., Tessier-Cloutier B., Praetorius T., Orr N.L., Noga H., Lum A., Khattra J. (2019). Iatrogenic endometriosis harbors somatic cancer-driver mutations. Hum. Reprod..

[22] Orr N.L., Albert A., Liu Y.D., Lum A., Hong J., Ionescu C.L., Senz J., Nazeran T.M., Lee A.F., Noga H. (2023). KRAS mutations and endometriosis burden of disease. J. Pathol. Clin. Res..

[23] Kurman R.J., Hedrick Ellenson L., Ronnett B.M. (2019). Blaustein’s Pathology of the Female Genital Tract.

[24] Noe M., Ayhan A., Wang T.L., Shih I.M. (2018). Independent development of endometrial epithelium and stroma within the same endometriosis. J. Pathol..

[25] Li L., Yue P., Song Q., Yen T.T., Asaka S., Wang T.L., Beavis A.L., Fader A.N., Jiao Y., Yuan G. (2021). Genome-wide mutation analysis in precancerous lesions of endometrial carcinoma. J. Pathol..

[26] Wu R.C., Wang P., Lin S.F., Zhang M., Song Q., Chu T., Wang B.G., Kurman R.J., Vang R., Kinzler K. (2019). Genomic landscape and evolutionary trajectories of ovarian cancer precursor lesions. J. Pathol..

[27] Tokheim C.J., Papadopoulos N., Kinzler K.W., Vogelstein B., Karchin R. (2016). Evaluating the evaluation of cancer driver genes. Proc. Natl. Acad. Sci. USA.

[28] Notzold A., Moubayed P., Sievers H.H. (1998). Endometriosis in the thoracic aorta. N. Engl. J. Med..

[29] Beavis A.L., Matsuo K., Grubbs B.H., Srivastava S.A., Truong C.M., Moffitt M.N., Maliglig A.M., Lin Y.G. (2011). Endometriosis in para-aortic lymph nodes during pregnancy: Case report and review of literature. Fertil. Steril..

[30] Centini G., Ginetti A., Colombi I., Cannoni A., Giorgi M., Ferreira H., Fedele F., Pacifici M., Martire F.G., Zupi E. (2024). Endometriosis of the appendix: Prevalence, associated lesions, and proposal of pathogenetic hypotheses. A retrospective cohort study with prospectively collected data. Arch. Gynecol. Obstet..

[31] Kandoth C., Schultz N., Cherniack A.D., Akbani R., Liu Y., Shen H., Robertson A.G., Pashtan I., Shen R., The Cancer Genome Atlas Research Network (2013). Integrated genomic characterization of endometrial carcinoma. Nature.

[32] Vogelstein B., Papadopoulos N., Velculescu V.E., Zhou S., Diaz L.A., Kinzler K.W. (2013). Cancer genome landscapes. Science.

[33] Ayhan A., Mao T.-L., Rahmanto Y.S., Zeppernick F., Ogawa H., Wu R.C., Wang T.L., Shih I.M. (2015). Increased proliferation in atypical hyperplasia/endometrioid intraepithelial neoplasia of the endometrium with concurrent inactivation of ARID1A and PTEN tumour suppressors. J. Patho Clin. Res..

[34] Suryo Rahmanto Y., Shen W., Shi X., Chen X., Yu Y., Yu Z.C., Miyamoto T., Lee M.H., Singh V., Asaka R. (2020). Inactivation of Arid1a in the endometrium is associated with endometrioid tumorigenesis through transcriptional reprogramming. Nat. Commun..

[35] Mandal J., Yu Z.C., Shih I.M., Wang T.L. (2023). ARID1A loss activates MAPK signaling via DUSP4 downregulation. J. Biomed. Sci..

[36] Dinulescu D.M., Ince T.A., Quade B.J., Shafer S.A., Crowley D., Jacks T. (2005). Role of K-ras and Pten in the development of mouse models of endometriosis and endometrioid ovarian cancer. Nat. Med..

[37] Cheng C.W., Licence D., Cook E., Luo F., Arends M.J., Smith S.K., Print C.G., Charnock-Jones D.S. (2011). Activation of mutated K-ras in donor endometrial epithelium and stroma promotes lesion growth in an intact immunocompetent murine model of endometriosis. J. Pathol..

[38] Yoo J.Y., Kim T.H., Fazleabas A.T., Palomino W.A., Ahn S.H., Tayade C., Schammel D.P., Young S.L., Jeong J.W., Lessey B.A. (2017). KRAS Activation and over-expression of SIRT1/BCL6 Contributes to the Pathogenesis of Endometriosis and Progesterone Resistance. Sci. Rep..

[39] Pandya D., Tomita S., Rhenals M.P., Swierczek S., Reid K., Camacho-Vanegas O., Camacho C., Engelman K., Polukort S., RoseFigura J. (2024). Mutations in cancer-relevant genes are ubiquitous in histologically normal endometrial tissue. Gynecol. Oncol..

[40] Bulun S.E. (2025). Endometriosis and ovulatory menstruation: Beyond the Sampson principle. J. Clin. Investig..

[41] Zhang Q., Zhu F., Tong Y., Shi D., Zhang J. (2025). CHD4 R975H mutant activates tumorigenic pathways and promotes stemness and M2-like macrophage polarization in endometrial cancer. Sci. Rep..

[42] Daniloski Z., Smith S. (2017). Loss of Tumor Suppressor *STAG2* Promotes Telomere Recombination and Extends the Replicative Lifespan of Normal Human Cells. Cancer Res..

